# Hypomagnesemia is not an independent risk factor for mortality in Japanese maintenance hemodialysis patients

**DOI:** 10.1007/s11255-019-02073-w

**Published:** 2019-04-11

**Authors:** Sonoo Mizuiri, Yoshiko Nishizawa, Kazuomi Yamashita, Takayuki Naito, Kyoka Ono, Chie Tanji, Koji Usui, Shigehiro Doi, Takao Masaki, Kenichiro Shigemoto

**Affiliations:** 1 Division of Nephrology, Ichiyokai Harada Hospital, 7-10 Kairoyama-cho, Saeki-ku, Hiroshima, 731-5134 Japan; 2 Ichiyokai Ichiyokai Clinic, 10-3 Asahien, Saeki-ku, Hiroshima, 731-5133 Japan; 3 Department of Nephrology, Hiroshima University Hospital, 1-2-3 Kasumi Minami-ku, Hiroshima, 734-8551 Japan

**Keywords:** Coronary artery calcium score, Hemodialysis, Hypomagnesemia, Malnutrition, Mortality

## Abstract

**Purpose:**

It is unclear whether hypomagnesemia is an independent risk factor or innocent bystander for mortality in maintenance hemodialysis (MHD) patients. Thus, we studied associations between hypomagnesemia and all-cause as well as cardiovascular (CV) mortality in MHD patients.

**Methods:**

Baseline clinical characteristics and coronary artery calcium score (CACS) of 353 Japanese MHD patients were reviewed. Three-year survival rate and mortality risk factors were assessed.

**Results:**

Median (interquartile range) age, dialysis vintage, serum magnesium (Mg), serum albumin and CACS of the subjects were 68 (60–78) years, 75 (32–151) months, 2.4 (2.2–2.7) mg/dl, 3.6 (3.3–3.8) g/dl, and 1181 (278–3190), respectively. During the 3-year period, 91 patients died. Kaplan–Meier overall 3-year survival rates were 59.0% in in patients with Mg < 2.4 mg/dl (*n* = 136) and 82.3% in patients with Mg ≥ 2.4 mg/dl (*n* = 217), (*P* < 0.0001). In Cox regression models not incorporating serum albumin, Mg < 2.4 mg/dl was significantly associated with 3-year all-cause death, independent of age, dialysis vintage, average ultrafiltration, Log (CACS + 1), warfarin use, serum potassium, high-sensitivity C-reactive protein (hsCRP), phosphate, uric acid, and intact parathyroid hormone [Hazard ratio (HR) 95% confidence interval (CI): 2.82 (1.31–6.29), *P* = 0.0078], and CV death, independent of age, dialysis vintage, Log (CACS + 1), warfarin use, serum hsCRP, and uric acid [HR (95% CI): 4.47 (1.45–16.76), *P* = 0.0086]. Nevertheless, associations of Mg < 2.4 mg/dl with all-cause and CV mortality were all absent in models that included serum albumin.

**Conclusions:**

Hypomagnesemia is not an independent risk factor for mortality but is associated with malnutrition in MHD patients.

## Introduction

Vascular calcification is common in hemodialysis (HD) patients and increases the risk of cardiovascular (CV) disease [1]. Recently, interest has grown in the relationships between serum magnesium (Mg) levels and vascular calcification or mortality in HD patients [2]. Extracellular magnesium accounts for only 1% of the total body content [3, 4]. Serum contains a much smaller amount (0.3% of total body stores), of which one-third is bound to protein, and nonprotein bound magnesium is the fraction that is ultrafilterable in HD and consists of complexed and free magnesium (5.5% and 61% of total serum levels, respectively) [4]. Normal Mg concentration is 1.7–2.4 mg/dl, and magnesium homeostasis is determined by the balance between absorption and excretion (intestinal and renal) [3, 4]. In experimental studies, magnesium inhibited vascular calcification and osteogenic differentiation in vitro [5], prevented calciprotein particle maturation, which can induce calcification in vitro [6], and bound to phosphate in the intestines, which reduced vascular calcification in rats [7]. In clinical studies, the follow-up periods used in previous large studies have been short [8, 9]; notably, few interventional studies have been performed [2, 10]. Clinical studies have produced inconsistent results regarding the association between Mg levels and mortality, partly because hypomagnesemia is linked to an increased frequency of co-morbidities. It is unclear whether hypomagnesemia causes increased mortality as an independent risk factor or as an “innocent bystander” [11].

This study was undertaken to investigate the associations between hypomagnesemia and all-cause as well as CV mortality in maintenance hemodialysis (MHD) patients.

## Materials and methods

This prospective observational study included 353 unselected patients that received MHD during September 2014 at Ichiyokai Hospitals. Patients who met any of the following criteria were excluded: age < 20 years old, had been on dialysis for < 3 months, had a history of advanced cancer, had experienced an infection in the past month, or had undergone organ transplantation. All patients underwent 4-h HD sessions, with high-flux membranes and standard bicarbonate dialysis fluid (140 mEq/l sodium, 2.0 mEq/l potassium, 3.0 mEq/l calcium, 1.0 mEq/l magnesium, and 100 mg/dl glucose) using central dialysis fluid delivery system, 3 times per week. Patients’ baseline characteristics, including information regarding age, gender, primary kidney disease, dialysis vintage, presence of diabetes mellitus, and current use of warfarin, were obtained from the institutional database. Blood pressure values, Kt/Vurea, Mg, serum albumin, high-sensitivity C-reactive protein (hsCRP), potassium, phosphate, uric acid, intact parathyroid hormone (iPTH), intact fibroblast growth factor 23 (FGF23), albumin-adjusted serum calcium levels, normalized protein nitrogen appearance (nPNA), and geriatric nutritional risk index (GNRI) were measured at baseline, just before or after (for Kt/Vurea alone) the first dialysis session of the first week in September 2014. Furthermore, 24-h urinary volume was evaluated 1 day before baseline blood sampling. Urine volume ≥ 100 ml/day was used as a surrogate marker for residual renal function; no residual renal function was defined as urine volume < 100 ml/day in this study. The average ultrafiltration/body weight before treatment in the first hemodialysis session of the week in September 2014 (ml/kg/h) was also evaluated. The coronary artery calcium score (CACS) was assessed using the Agatston score [12], which was obtained from thoracoabdominal multi-detector computed tomography with an Aquilion 64 TSX-101A (Toshiba Medical Systems, Tokyo, Japan). The all-cause and CV mortalities during the follow-up period (from September 2014 to September 2017) were confirmed based on documentation. The CV mortality rate was calculated based on deaths due to CV disease (coronary artery disease, aortic aneurysms, cerebral infarction, cerebral hemorrhage, and/or peripheral artery disease). Patients were followed until death or the end of the study (for 1095 days); their Mg and serum hsCRP levels were determined by BML, Inc. (Tokyo, Japan). A colorimetric method for quantitative Mg determination (Clonemate MG reagent; Sekisui Medical, Tokyo, Japan) was used, which has a normal range of 1.7 to 2.4 mg/dl in the general population and a coefficient of variation of approximately 0.83–1.71%. Serum FGF23 levels were determined by SRL, Inc. (Tokyo, Japan), which used a sandwich enzyme-linked immunosorbent assay kit (Kainos Laboratories, Tokyo, Japan). Our hospital’s laboratory performed the remaining clinical biochemical analyses.

### Statistical analysis

All statistical analyses were performed with JMP 13 (SAS Institute Japan, Tokyo, Japan). Data for categorical variables are shown as numbers of patients (percentages); data for continuous variables are shown as mean ± standard deviation (SD) or median values and interquartile range (IQR), as appropriate. The significance of inter-group differences was analyzed with the Kruskal–Wallis test, or *χ*^2^ test, as appropriate. Kaplan–Meier 3-year cumulative survival for all-cause deaths and CV deaths was compared between patients with Mg level below the median value and patients with Mg level above the median value in all subjects and subgroups according to serum albumin quartiles. Hazard ratios (HRs) for all-cause and CV death, with associated 95% confidence intervals (CIs), were calculated with the use of a stratified Cox proportional-hazards model. Univariate and multivariate Cox proportional-hazards models were used to determine the factors associated with 3-year all-cause and CV mortality. The distributions of FGF23 and CACS were markedly skewed. Prior to the Cox proportional hazards analyses, FGF23 was transformed to Log FGF23 and CACS was transformed to Log (CACS + 1), because some of the study participants had a CACS of 0.

## Results

The MHD patients’ primary diseases were as follows: diabetic nephropathy (*n* = 142, 40.2%), chronic glomerulonephritis (*n* = 121, 34.3%), nephrosclerosis (*n* = 43, 12.2%), polycystic kidney disease (*n* = 11, 3.1%), other diseases (*n* = 13, 3.7%), and unknown conditions (*n* = 23, 6.5%). The baseline characteristics of the patients are shown in Table 1. Among all patients (*n* = 353), the median (IQR) age was 68 (60–78) years and 66.6% were male. The median (IQR) dialysis vintage was 75 (32–151) months. The median (IQR) Mg level was 2.4 (2.2–2.7) mg/dl; four (1.1%) patients had Mg < 1.7 mg/dl, 179 (50.7%) patients had Mg values in the normal range for the general population (1.7–2.4 mg/dl), and 170 (48.2%) patients had Mg > 2.4 mg/dl. The same Mg values were observed in many patients in this study, and the groups were not correctly divided into quartiles; notably, the four groups according to Mg differed considerably in the numbers of patients. Mg levels were < 2.2 mg/dl, 2.2–2.4 mg/dl, 2.5–2.7 mg/dl, and > 2.7 mg/dl in group (G)1 (*n* = 86), G2 (*n* = 97), G3 (*n* = 99), and G4 (*n* = 71), respectively. Our study showed that age, dialysis vintage, average ultrafiltration, frequency of warfarin use, serum potassium, hsCRP, phosphate, uric acid, albumin, and FGF23 levels were significantly different among the four groups (*P* < 0.05). There were also significant differences in nPNA [0.80 (0.60–0.92), 0.89 (0.78–1.04), 0.94 (0.83–1.05), and 0.97 (0.85–1.11) g/kg/day, *P* < 0.0001], GNRI [89 (80–94), 92 (86–97), 94 (91–98), and 95 (89–98), *P* < 0.0001], Kt/Vurea [1.30 (1.12–1.46), 1.40 (1.25–1.56), 1.41 (1.30–1.62), and 1.42 (1.26–1.57)/session, *P* = 0.0003], and diastolic blood pressure [77 ± 15, 79 ± 14, 82 ± 13, and 83 ± 14 mmHg, *P* = 0.0003] in G1, G2, G3 and G4 (data not shown). No significant differences were detected among the four groups in the frequency of male gender, frequency of diabetes mellitus, frequency of urine volume ≥ 100 ml/day, CACS, or iPTH values. Furthermore, no significant differences were detected in albumin-adjusted serum calcium [9.4 (8.9–10.0), 9.3 (8.9–9.8), 9.5 (9.0–10.0), and 9.4 (9.1–10.1) mg/dl, *P* = 0.5318] and systolic blood pressure [147 ± 25, 146 ± 23, 150 ± 20, and 154 ± 22 mmHg, *P* = 0.2221] in G1, G2, G3 and G4 (data not shown).

**Table 1:** Baseline characteristics of maintenance hemodialysis patients (*n* = 353), stratified by the serum magnesium value

Characteristics	All	Group 1	Group 2	Group 3	Group 4	*P*
		(Mg < 2.2 mg/dl)	(Mg 2.2–2.4 mg/dl)	(Mg 2.5–2.7 mg/dl)	(Mg > 2.7 mg/dl)	
	*n* = 353	*n* = 86	*n* = 97	*n* = 99	*n* = 71	
Serum magnesium (mg/dl)	2.4 (2.2–2.7)	2.0 (1.9–2.1)	2.3 (2.2–2.4)	2.6 (2.5–2.7)	2.9 (2.8–3.1)	< 0.0001
Age (years)	68 (60–78)	73 (64–80)	71 (62–79)	66 (59–73)	63 (55–73)	< 0.0001
Gender (male), *n* (%)	235/353 (66.6)	62/86 (72.1)	62/97 (63.9)	66/99 (66.7)	45/71 (63.4)	0.6126
Dialysis vintage (months)	75 (32–151)	55 (23–105)	60 (28–126)	85 (46–175)	93 (48–183)	0.0044
Diabetes mellitus, *n* (%)	142/353 (40.2)	41/86 (47.7)	37/97 (38.1)	36/99 (36.7)	28/71 (39.4)	0.4454
Urine volume ≥ 100 ml/day, *n* (%)	87/353 (24.6)	20/86 (23.2)	31/97 (32.0)	21/99 (21.2)	15/71 (21.1)	0.2631
Average ultrafiltration (ml/kg/h)	11.2 ± 3.1	10.4 ± 3.5	11.0 ± 2.8	11.7 ± 2.6	11.8 ± 3.5	0.0122
CACS	1181 (278–3190)	1550 (318–3715)	1145 (254–2872)	1030 (247–2520)	1057 (257–2357)	0.5483
Warfarin use, *n* (%)	39/353 (11.0)	15/86 (17.4)	4/97 (4.1)	15/99 (15.1)	5/71 (7.0)	0.0109
Serum potassium (mEq/l)	4.7 ± 0.8	4.3 ± 0.8	4.6 ± 0.7	5.0 ± 0.7	5.0 ± 0.8	< 0.0001
Serum hsCRP (mg/dl)	0.12 (0.04–0.43)	0.26 (0.06–0.80)	0.12 (0.03–1.49)	0.12 (0.03–0.35)	0.07 (0.03–0.19)	< 0.0008
Serum phosphate (mg/dl)	5.1 (4.1–6.0)	4.2 (3.2–5.4)	5.2 (4.2–6.1)	5.3 (4.7–6.3)	5.1 (4.4–6.1)	< 0.0001
Serum uric acid (mg/dl)	7.3 ± 1.4	6.6 ± 1.4	7.4 ± 1.4	7.4 ± 1.1	7.6 ± 1.5	< 0.0001
Serum intact parathyroid hormone (pg/ml)	109 (51–208)	107 (49–183)	110 (57–210)	128 (60–241)	97 (38–183)	0.1247
Serum albumin (g/dl)	3.6 (3.3–3.8)	3.3 (2.9–3.6)	3.6 (3.3–3.8)	3.6 (3.4–3.8)	3.7 (3.5–3.9)	< 0.0001
FGF23 (pg/ml)	3165 (590–11075)	784 (247–5510)	3210 (769–11650)	4470 (1305–13425)	4680 (1150–11500)	< 0.0001

Mg, serum magnesium; Diabetes mellitus, presence of diabetes mellitus; Average ultrafiltration (ml/kg/h), average ultrafiltration/body weight before treatment in the first hemodialysis session of the week in September 2014 (ml/kg/h); CACS, coronary artery calcium score; Warfarin use, current use of warfarin; hsCRP, high-sensitivity C-reactive protein; FGF23, intact fibroblast growth factor 23

**Fig. 1:**
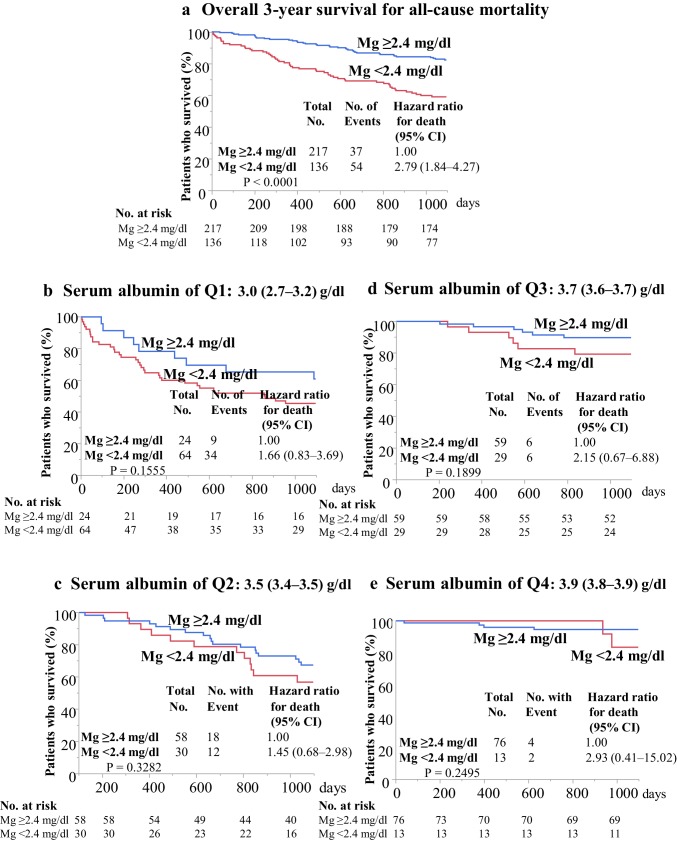
Kaplan–Meier overall 3-year survival for all-cause death (**a**) and 3-year survival for all-cause death in subgroups according to serum albumin quartiles (**b**–**e**). Overall survival was worse in patients with Mg values below the median value (< 2.4 mg/dl, *n* = 136) than in patients with Mg values above the median value (≥ 2.4 mg/dl, *n* = 217). The hazard ratio for death (95% confidence interval) was 2.79 (1.84–4.27), *P* < 0.0001 (**a**). However, the significant difference was lost in subgroup analyses according to serum albumin quartiles. Mg, serum magnesium; No., numbers; CI, confidence interval

Ninety-one patients died during the 3-year observation period, and the 3-year cumulative survival rate in all subjects was 73.3%. CV disease (51.6%, 47/91) was the primary cause of death, followed by infection (30.8%, 28/91), malignancy (3.3%, 3/91), other causes (11.0%, 10/91), and unknown conditions (3.3%, 3/91). Kaplan–Meier plots showed that 3-year overall survival for all-cause mortality was 59.0% in patients with Mg value below the median value (< 2.4 mg/dl, *n* = 136) and 82.3% in patients with Mg value above the median value (≥ 2.4 mg/dl, *n* = 217). The HR (95% CI) for 3-year all-cause death in patients with Mg < 2.4 mg/dl was 2.79 (1.84–4.27), *P* < 0.0001 (Fig. 1a). However, 3-year cumulative survival for all-cause mortality in patients with Mg < 2.4 mg/dl did not differ significantly from that of patients with Mg ≥ 2.4 mg/dl in all subgroups that were analyzed, including those with serum albumin of quartile (Q)1 [3.0 (2.7–3.2) g/dl, *n* = 88], serum albumin of Q2 [3.5 (3.4–3.5) g/dl, *n* = 88], serum albumin of Q3 [3.7 (3.6–3.7) g/dl, *n* = 88], and serum albumin of Q4 [3.9 (3.8–3.9) g/dl, *n* = 89] (Fig. 1b–e).

**Fig. 2:**
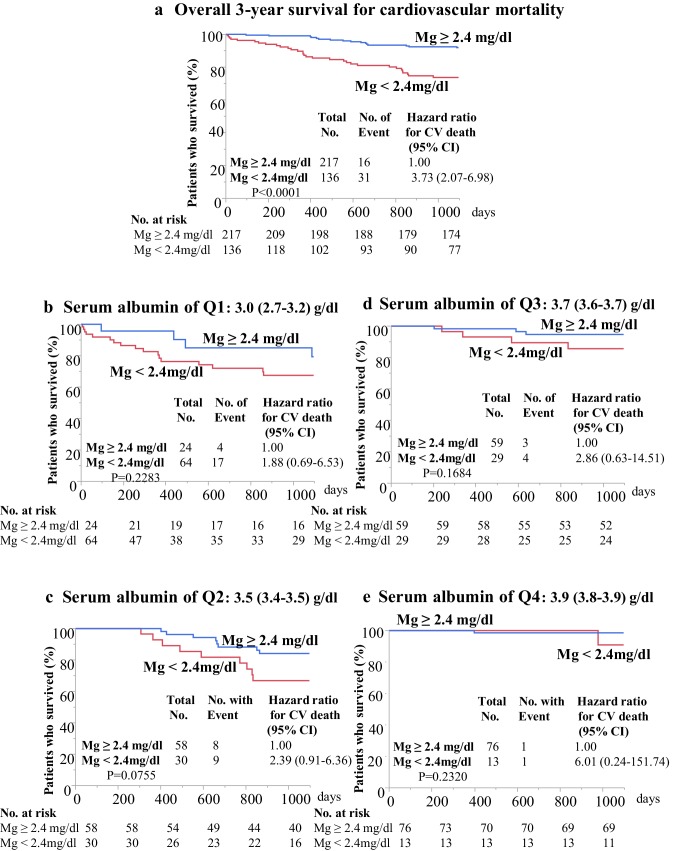
Kaplan–Meier overall 3-year survival for cardiovascular (CV) mortality (**a**) and 3-year survival for CV mortality in subgroups according to serum albumin quartiles (**b**–**e**). Overall survival for CV death was worse in patients with Mg values below the median value (< 2.4 mg/dl, *n* = 136) than in patients with Mg values greater than the median value (≥ 2.4 mg/dl, *n* = 217). The hazard ratio (95% confidence interval) for CV death was 3.73 (2.07–6.98), *P* < 0.0001 (**a**). However, the significant difference for CV mortality was lost in subgroup analyses when serum albumin quartiles were incorporated (**b**–**e**). Mg, serum magnesium; No., numbers; CI, confidence interval

Kaplan–Meier plots also showed significantly worse overall 3-year survival for CV mortality in patients with Mg < 2.4 mg/dl (*n* = 136), compared with that in patients with Mg ≥ 2.4 mg/dl (*n* = 217); the HR (95% CI) for 3-year CV death in patients with Mg < 2.4 mg/dl was 3.73 (2.07–6.98), *P* < 0.0001(Fig. 2a). However, 3-year cumulative survival for CV mortality in patients with Mg < 2.4 mg/dl did not differ significantly from that of patients with Mg ≥ 2.4 mg/dl in all subgroups that were analyzed, including those with serum albumin of Q1, Q2, Q3, and Q4 (Fig. 2b–e).

The predictors of 3-year all-cause mortality in MHD patients by Cox proportional hazard analyses are shown in Table 2. They included the age, dialysis vintage, average ultrafiltration, Log (CACS + 1), warfarin use, serum potassium, hsCRP, phosphate, uric acid, iPTH, Mg < 2.4 mg/dl, serum albumin, gender (male), presence of diabetes mellitus, urine volume ≥ 100 ml/day, and Log FGF23 at baseline. In the univariate analyses, all variables except gender (male), presence of diabetes mellitus, urine volume ≥ 100 ml/day, and Log FGF23 were significantly associated with 3-year all-cause mortality (*P* < 0.05). In multivariate analyses, Mg < 2.4 mg/dl was a significant all-cause mortality risk factor independent of age, dialysis vintage, average ultrafiltration, Log (CACS + 1), warfarin use, serum potassium, hsCRP, phosphate, uric acid, and iPTH in model 1 [HR (95% CI) 2.82 (1.31–6.29), *P* = 0.0078]. However, the statistical significance of Mg < 2.4 mg/dl was lost in model 2, which included the same variables as model 1 plus serum albumin.

**Table 2:** Predictors of 3-year all-cause mortality in maintenance hemodialysis patients, according to Cox proportional hazard analyses (*n* = 353)

Independent variable	Univariate analyses			Multivariate analyses					
				Model 1			Model 2		
	HR	95% CI	*P*	HR	95% CI	*P*	HR	95% CI	*P*
Age (years)	1.08	1.06–1.11	< 0.0001	1.07	1.03–1.12	0.0002	1.06	1.01–1.10	0.0088
Dialysis vintage (months)	1.00	0.99–1.00	< 0.0091	1.00	0.99–1.00	0.5028	1.00	0.99–1.00	0.8750
Average ultrafiltration (ml/kg/h)	0.93	0.86–0.99	0.0295	0.99	0.87–1.13	0.8700	0.98	0.87–1.12	0.8109
Log (CACS + 1)	2.07	1.24–3.84	0.0036	1.74	1.01–3.26	0.0472	1.99	1.03–3.84	0.0246
Warfarin use	2.51	1.47–4.08	0.0012	3.88	1.75–8.17	0.0013	4.52	2.03–9.54	0.0004
Serum potassium (mEq/l)	0.60	0.45–0.79	0.0002	1.28	0.70–2.31	0.4172	1.53	0.81–2.87	0.1878
Serum hsCRP (mg/dl)	1.57	1.39–1.77	< 0.0001	1.20	0.88–1.57	0.2257	1.12	0.84–1.49	0.4623
Serum phosphate (mg/dl)	0.73	0.63–0.84	< 0.0001	1.07	0.78–1.46	0.6795	1.17	0.86–1.60	0.3191
Serum uric acid (mg/dl)	0.63	0.53–0.75	< 0.0001	0.79	0.55–1.10	0.1658	0.87	0.62–1.22	0.4152
Intact parathyroid hormone (pg/ml)	1.00	0.99–1.00	0.0357	0.99	0.99–1.00	0.9694	1.00	0.99–1.00	0.8527
Serum Mg < 2.4 mg/dl	2.79	1.84–4.27	< 0.0001	2.82	1.31–6.29	0.0078	2.17	0.96–5.02	0.0633
Serum albumin (g/dl)	0.13	0.10–0.19	< 0.0001				0.21	0.09–0.49	0.0007
Gender (male)	0.87	0.57–1.35	0.5262						
Diabetes mellitus	1.39	0.92–2.09	0.1205						
Urine volume ≥ 100 ml/day	0.67	0.39–1.10	0.1136						
Log FGF23 (pg/ml)	1.00	0.99–1.00	0.1086						

Model 1 included age, dialysis vintage, average ultrafiltration, Log (CACS + 1), warfarin use, serum potassium, hsCRP, phosphate, uric acid, intact parathyroid hormone, and serum Mg < 2.4 mg/dl, which exhibited significance in the univariate analyses; serum albumin was excluded. Model 2 included the same variables as model 1 plus serum albumin

Mg, magnesium; Diabetes mellitus, presence of diabetes mellitus; Average ultrafiltration (ml/kg/h), average ultrafiltration/body weight before treatment in the first hemodialysis session of the week in September 2014 (ml/kg/h); CACS, coronary artery calcium score; Warfarin use, current use of warfarin; hsCRP, high-sensitivity C-reactive protein; FGF23, intact fibroblast growth factor 23; HR, hazard ratio; CI, confidence interval

The predictors of 3-year CV mortality identified in MHD patients by Cox proportional hazard analyses are shown in Table 3. The same independent variables in Table 2 were included in the univariate Cox models. In the univariate analyses, the age, dialysis vintage, Log (CACS + 1), warfarin use, serum hsCRP, uric acid, Mg < 2.4 mg/dl, and serum albumin were found to be significantly associated with 3-year CV mortality (*P* < 0.05). However, other variables, including the gender (male), presence of diabetes mellitus, urine volume ≥ 100 ml/day, average ultrafiltration, serum potassium, phosphate, iPTH, and Log FGF23, were not. Multivariate analyses showed that Mg < 2.4 mg/dl was significantly associated with 3-year CV mortality, independent of age, dialysis vintage, Log (CACS + 1), warfarin use, serum hsCRP, and uric acid in model 1 [HR (95% CI): 4.47 (1.45–16.76), *P* = 0.0086]. However, the statistical significance of Mg < 2.4 mg/dl was lost in model 2, which included the same variables as model 1 plus serum albumin.

**Table 3:** Predictors of 3-year cardiovascular mortality in maintenance hemodialysis patients, according to Cox proportional hazard analyses (*n* = 353)

Independent variable	Univariate analyses			Multivariate analyses					
				Model 1			Model 2		
	HR	95% CI	*P*	HR	95% CI	*P*	HR	95% CI	*P*
Age (years)	1.08	1.05–1.11	< 0.0001	1.09	1.03–1.18	0.0058	1.08	1.01–1.17	0.0251
Dialysis vintage (months)	1.00	0.99–1.00	0.0091	1.00	1.00–1.01	0.5429	1.00	0.99–1.01	0.8450
Log (CACS + 1)	2.94	1.21–9.21	0.0126	2.46	1.03–7.27	0.0416	2.79	1.06–9.06	0.0372
Warfarin use	2.93	1.42–5.58	0.0049	6.21	1.95–19.13	0.0029	7.27	2.22–23.04	0.0017
Serum hsCRP (mg/dl)	1.57	1.29–1.84	< 0.0001	1.06	0.65–1.50	0.7924	1.03	0.58–1.40	0.8834
Serum uric acid (mg/dl)	0.69	0.54–0.87	0.0012	1.18	0.81–1.73	0.3979	1.50	0.99–2.33	0.0568
Serum Mg < 2.4 mg/dl	3.73	2.07–6.98	< 0.0001	4.47	1.45–16.76	0.0086	2.56	0.72–10.36	0.1485
Serum albumin (g/dl)	0.15	0.09–0.24	< 0.0001				0.15	0.04–0.57	0.0062
Gender (male)	0.83	0.46–1.54	0.5424						
Diabetes mellitus	1.47	0.83–2.62	0.1859						
Urine volume ≥ 100 ml/day	0.87	0.48–1.68	0.6725						
Average ultrafiltration (ml/kg/h)	0.94	0.85–1.03	0.1947						
Serum potassium (mEq/l)	0.69	0.47–1.01	0.0565						
Serum phosphate (mg/dL)	0.93	0.76–1.14	0.4977						
Intact parathyroid hormone (pg/ml)	1.00	0.99–1.00	0.5725						
Log FGF23 (pg/ml)	1.00	0.99–1.00	0.3003						

Model 1 included age, dialysis vintage, Log (CACS + 1), warfarin use, serum hsCRP, uric acid, and serum Mg < 2.4 mg/dl, which exhibited significance in the univariate analyses; serum albumin was excluded. Model 2 included the same variables as model 1 plus serum albumin

Mg, magnesium; Diabetes mellitus, presence of diabetes mellitus; Average ultrafiltration (ml/kg/h), average ultrafiltration/body weight before treatment in the first hemodialysis session of the week in September 2014 (ml/kg/h); CACS, coronary artery calcium score; Warfarin use, current use of warfarin; hsCRP, high-sensitivity C-reactive protein; FGF23, intact fibroblast growth factor 23; HR, hazard ratio; CI, confidence interval

## Discussion

The estimation of total body magnesium stores based on serum levels can be problematic as this fraction is not necessarily readily exchangeable with other body compartments. However, the most common method of clinical assessment of magnesium balance remains the total Mg [4]. Recently, there has been an increasing number of clinical reports regarding the associations between Mg levels and mortality and CV mortality in HD patients. Most studies found that lower Mg levels were associated with increased all-cause and CV mortality [9, 13–15]. However, Ishimura et al. reported that all-cause and non-CV mortality (but not CV mortality) were associated with lower Mg levels [16]. Our study showed worse 3-year cumulative overall survival for all-cause and CV death in patients with hypomagnesemia (Mg < 2.4 mg/dl); however, the significance was lost when serum albumin quartile was incorporated in the models. Cox proportional hazard analyses in our study showed that Mg < 2.4 mg/dl was a significant 3-year all-cause mortality risk factor, independent of age, dialysis vintage, average ultrafiltration, Log (CACS + 1), warfarin use, serum potassium, hsCRP, phosphate, uric acid, and iPTH. Furthermore, Mg < 2.4 mg/dl was a significant predictor of 3-year CV death, independent of age, dialysis vintage, Log (CACS + 1), warfarin use, serum hsCRP, and uric acid. However, upon inclusion of serum albumin in the models, the all-cause and CV mortality risk prediction of hypomagnesemia was lost. This indicates that hypomagnesemia alone was not an independent predictor of all-cause and CV mortality, but was associated with malnutrition. The median (IQR) age was 68 (60–78) years and the 3-year cumulative survival rate was 73.3% in our subjects; these were consistent with the 3-year cumulative survival rate (72.9%) in Japanese MHD patients for a similar median age [17]. This seems relevant for generalization of the results, at least in Japanese patients.

A cohort study of 142,555 Japanese HD patients by Sakaguchi et al. [8] and a study of 206 HD patients by Matias et al. [13] revealed that hypomagnesemia is a significant mortality risk factor, independent of malnutrition; this is inconsistent with our results. Notably, all three of those studies were prospective observational studies; the observational period was 1–2 years in those studies and 3 years in our study. It has been reported that MHD patients tend to exhibit higher Mg levels than healthy controls [4, 18]. The frequency of hypermagnesemia (> 2.4 mg/dl) in our study was 170/353 (48.2%), which is lower than the frequencies reported by Sakaguchi et al. [87,993/142,555 (61.7%)] [8] or Matias et al. [197/206 (95.5%)] [13]. The different results in these studies may be explained by changes in magnesium balance in the analyzed patients, perhaps due to low food intake, processed foods with a low magnesium content in patients, and low dialysate magnesium concentration (1 mEq/l) in Japan, compared with the dialysate used by Matias et al. (2.0 mEq/l). There is a possibility that the lower frequency of hypermagnesemia in our study may mask some positive effect of Mg in our cohort. Sakaguchi et al. detected a J-shaped relationship between Mg levels and mortality, in which Mg levels of < 2.7 mg/dl and ≥ 3.1 mg/dl were associated with increased risks of all-cause mortality [8]. Optimal Mg concentration in HD patients may be above the reference range for the general population. We agree with the suggestion by Sakaguchi et al. that it is important to determine both the lower and upper limits of the target range of Mg in HD patients [8], because hypermagnesemia has been linked to osteomalacia; moreover, activation of the calcium-sensing receptor results in lower PTH levels [3, 19]. Further studies are needed to determine optimal Mg levels and dialysate magnesium concentrations for HD patients.

It has been reported that lower Mg levels are associated with reduced serum phosphorus, calcium, and albumin levels; thus, they might be indicative of malnutrition [9, 16, 20]. It has also been reported that lower Mg levels are linked with poorer nutritional status and increased inflammation [21]. A large study found that hypomagnesemia (< 1.9 mg/dl) conferred an increased risk of mortality solely in the presence of low-serum albumin levels (< 35 g/l) [22]. In our study, MHD patients with Mg < 2.4 mg/dl had worse nutritional status, which comprised lower values of serum albumin, nPNA, GNRI, phosphate, and uric acid, compared with patients with Mg ≥ 2.4 mg/dl. Taken together with the results of the previous reports [9, 16, 20–22], our findings indicate that nutritional factors greatly influence hypomagnesemia, and that such factors might partly contribute to the poor outcomes described by Courivaud et al. [11]. Thus, hypomagnesemia appears to be correlated with mortality; whether Mg plays a causal role in this relationship remains unclear [4, 11].

The presence of diabetes mellitus, FGF23 levels, and existing residual renal function were not significant predictors for 3-year all-cause and CV mortality in MHD patients in our study. We cannot completely exclude relevant bias in the analyses, but we have contemplated our results as below. The reported 5-year cumulative survival rates of HD patients with and without diabetes were 47% versus 31%, while corresponding 10-year cumulative survival rates were 22% versus 31% [*P* = 0.03]; the paths of the two groups were divergent at 5 years, suggesting that patients with diabetes may experience early mortality [23]. Early mortality in diabetic HD patients may be partly explained by the lack of an association between the presence of diabetes mellitus and mortality in our study, because our subjects were not incident HD patients and the median (IQR) dialysis vintage was 75 (32–151) months. The association of serum FGF23 levels and mortality in HD patients has been frequently described [24, 25]; however, no association was reported by Olauson et al. [26], which is consistent with our results. Discrepancies between these studies might be partly explained by the contributions of previous CV diseases, residual renal function levels, variations in the prevalence of diabetes [27], and racial differences. Urine volume ≥ 100 ml/day was used as a surrogate marker for residual renal function in this study, as in previous reports [28, 29]. An association between residual renal function (urine volume ≥ 100 ml/day) and mortality in MHD patients was not observed in our study, in contrast to previous reports [28, 29]; this may be related to the lower frequency of patients with residual renal function in our study [87/353 (24.6%) patients].

Our data did not show a significant difference of CACS between patients with Mg < 2.4 mg/dl and patients with Mg ≥ 2.4 mg/dl, in contrast to previous experimental studies [5–7]. However, we suspect that the lack of a cross-sectional association does not exclude a potential impact of persistently low Mg levels on CACS progression.

This study has several limitations. First, the study was a single analysis of laboratory values only at baseline. Therefore, there was no time-averaged analysis of laboratory data, and the influence of a single serum sample of magnesium, FGF23, or potassium value on 3-year all-cause and CV mortality might exhibit wide variation. Second, because the subjects were all Japanese MHD patients, the results might not be applicable to other populations. Third, the sample size and number of deaths were relatively small; therefore, the Cox regression analyses were restricted to a limited number of potential confounders. Fourth, there was no information regarding oral magnesium intake among these patients.

In conclusion, MHD patients with hypomagnesemia showed significantly worse 3-year cumulative survival for all-cause and CV death. Importantly, hypomagnesemia was not an independent risk factor for all-cause and CV mortality, but was associated with malnutrition in MHD patients.

## References

[1] Hamano N, Komaba H, Fukagawa M (2017). Magnesium as a new player in CKD: too little is as bad as too much?. Kidney Int.

[2] Sakaguchi Y, Hamano T, Isaka Y (2018). Magnesium in hemodialysis patients: a new understanding of the old problem. Contrib Nephrol.

[3] Alhosaini M, Leehey DJ (2015). Magnesium and dialysis: the neglected cation. Am J Kidney Dis.

[4] Misra PS, Nessim SJ (2017). Clinical aspects of magnesium physiology in patients on dialysis. Semin Dial.

[5] Montezano AC, Zimmerman D, Yusuf H, Burger D, Chignalia AZ, Wadhera V, van Leeuwen FN, Touyz RM (2010). Vascular smooth muscle cell differentiation to an osteogenic phenotype involves TRPM7 modulation by magnesium. Hypertension.

[6] Pasch A, Farese S, Gräber S, Wald J, Richtering W, Floege J, Jahnen-Dechent W (2012). Nanoparticle-based test measures overall propensity for calcification in serum. J Am Soc Nephrol.

[7] Diaz-Tocados JM, Peralta-Ramirez A, Rodríguez-Ortiz ME (2017). Dietary magnesium supplementation prevents and reverses vascular and soft tissue calcifications in uremic rats. Kidney Int.

[8] Sakaguchi Y, Fujii N, Shoji T, Hayashi T, Rakugi H, Isaka Y (2014). Hypomagnesemia is a significant predictor of cardiovascular and non-cardiovascular mortality in patients undergoing hemodialysis. Kidney Int.

[9] Lacson E, Wang W, Ma L, Passlick-Deetjen J (2015). Serum magnesium and mortality in hemodialysis patients in the United States: a cohort study. Am J Kidney Dis.

[10] Schmaderer C, Braunisch MC, Suttmann Y, Lorenz G, Pham D, Haller B, Angermann S, Matschkal J, Renders L, Baumann M, Braun JR, Heemann U, Küchle C (2017). Reduced mortality in maintenance haemodialysis patients on high versus low dialysate magnesium. A pilot study. Nutrients.

[11] Courivaud C, Davenport A (2014). Magnesium and the risk of all-cause and cardiac mortality in hemodialysis patients: agent provocateur or innocent bystander?. Kidney Int.

[12] Agatston AS, Janowitz WR, Hildner FJ, Zusmer NR, Viamonte M, Detrano R (1990). Quantification of coronary artery calcium using ultrafast computed tomography. J Am Coll Cardiol.

[13] João Matias P, Azevedo A, Laranjinha I, Navarro D, Mendes M, Ferreira C, Amaral T, Jorge C, Aires I, Gil C, Ferreira A (2014). Lower serum magnesium is associated with cardiovascular risk factors and mortality in haemodialysis patients. Blood Purif.

[14] devan RoijZuijdewijn CL, Grooteman MP, Bots ML, Blankestijn PJ, Steppan S, Büchel J, Groenwold RH, Brandenburg V, van den Dorpel MA, Ter Wee PM, Nubé MJ, Vervloet MG (2015). Serum magnesium and sudden death in European hemodialysis patients. PLoS ONE.

[15] Selim GN, Spasovski G, Tozija L, Georgievska-Ismail L, Zafirova-Ivanovska B, Masin-Spasovska J, Rambabova-Busletic I, Petronijevic Z, Dzekova-Vidimliski P, Ristovska V, Pusevski V, Stojceva-Taneva O (2017). Hypomagnesemia and cause-specific mortality in hemodialysis patients: 5-year follow-up analysis. Int J Artif Organs.

[16] Ishimura E, Okuno S, Kitatani K, Tsuchida T, Yamakawa T, Shioi A, Inaba M, Nishizawa Y (2007). Significant association between the presence of peripheral vascular calcification and lower serum magnesium in hemodialysis patients. Clin Nephrol.

[17] Masakane I, Nakai S, Ogata S, Kimata N, Hanafusa N, Hamano T, Wakai K, Wada A, Nitta K (2015). An overview of regular dialysis treatment in Japan (As of 31 December 2013). Ther Apher Dial.

[18] Navarro-González JF (1998). Magnesium in dialysis patients: serum levels and clinical implications. Clin Nephrol.

[19] Gonella M, Ballanti P, Della Rocca C, Calabrese G, Pratesi G, Vagelli G, Mazzotta A, Bonucci E (1988). Improved bone morphology by normalizing serum magnesium in chronically hemodialyzed patients. Miner Electrolyte Metab.

[20] Liu F, Zhang X, Qi H, Wang J, Wang M, Zhang Y, Yan H, Zhuang S (2013). Correlation of serum magnesium with cardiovascular risk factors in maintenance hemodialysis patients-a cross-sectional study. Magnes Res.

[21] Fein P, Suda V, Borawsky C, Kapupara H, Butikis A, Matza B, Chattopadhyay J, Avra MM (2010). Relationship of serum magnesium to body composition and inflammation in peritoneal dialysis patients. Adv Perit Dial.

[22] Li L, Streja E, Rhee CM, Mehrotra R, Soohoo M, Brunelli SM, Kovesdy CP, Kalantar-Zadeh K (2015). Hypomagnesemia and mortality in incident hemodialysis patients. Am J Kidney Dis.

[23] Browne OT, Allgar V, Bhandari S (2014). Analysis of factors predicting mortality of new patients commencing renal replacement therapy 10 years of follow-up. BMC Nephrol.

[24] Gutiérrez OM, Mannstadt M, Isakova T, Rauh-Hain JA, Tamez H, Shah A, Smith K, Lee H, Thadhani R, Jüppner H, Wolf M (2008). Fibroblast growth factor 23 and mortality among patients undergoing hemodialysis. N Engl J Med.

[25] Jean G, Terrat JC, Vanel T, Hurot JM, Lorriaux C, Mayor B, Chazot C (2009). High levels of serum fibroblast growth factor (FGF)-23 are associated with increased mortality in long haemodialysis patients. Nephrol Dial Transplant.

[26] Olauson H, Qureshi AR, Miyamoto T, Barany P, Heimburger O, Lindholm B, Stenvinkel P, Larsson TE (2010). Relation between serum fibroblast growth factor-23 level and mortality in incident dialysis patients: are gender and cardiovascular disease confounding the relationship?. Nephrol Dial Transplant.

[27] Kojima F, Uchida K, Ogawa T, Tanaka Y, Nitta K (2008). Plasma levels of fibroblast growth factor-23 and mineral metabolism in diabetic and non-diabetic patients on chronic hemodialysis. Int Urol Nephrol.

[28] Shemin D, Bostom AG, Laliberty P, Dworkin LD (2001). Residual renal function and mortality risk in hemodialysis patients. Am J Kidney Dis.

[29] Lee MJ, Park JT, Park KS, Kwon YE, Oh HJ, Yoo TH, Han SH, Kim YL, Kim YS, Yang CW, Kim NH, Kang SW (2017). Prognostic value of residual urine volume, GFR by 24-hour urine collection, and eGFR in patients receiving dialysis. Clin J Am Soc Nephrol.

